# Survey of MaineHealth Cancer Care Network Providers on Cannabis Use: Preparation for Studies Sponsored by the National Cancer Institute

**DOI:** 10.46804/2641-2225.1245

**Published:** 2026-01-09

**Authors:** Jill M Prescott, Jamie G Saunders, Leslie S Bradford, Scot C Remick

**Affiliations:** a MaineHealth Institute for Research, Scarborough, Maine; b Department of Obstetrics and Gynecology, MaineHealth Maine Medical Center, Portland, Maine

**Keywords:** Cancer, Cannabis, Clinical trials, Physician-patient relations, Survey

## Abstract

**Introduction:**

Cannabis and cannabinoid use in patients with cancer has rapidly scaled up over the past decade and is a topic of considerable clinical, research, and public health interest.

**Methods:**

We conducted a cannabis landscape survey among front-line providers in the MaineHealth Cancer Care Network (MHCCN) before applying and participating in National Cancer Institute (NCI)-sponsored studies on cannabis use in patients with cancer. The results of the survey can better inform participation in national studies and provide a departure point for provider educational activities focused on cannabis use in the cancer care setting.

**Results:**

Notable observations from our survey included a 58% (100/171) response rate, which signals awareness and interest in cannabis use by our provider teams. Also, 30% of providers/care team members inquire about their patients’ cannabis use, 89% agree that cannabis can be effective for managing symptoms, 54% are sensitive to stigma surrounding cannabis use (as well as 57% of their patients), only 15% considered themselves knowledgeable, and 85% are receptive to learning more about cannabis use.

**Discussion:**

These observations confirmed interest among our care teams to engage in cannabis-focused studies. They also paved a way toward participating in NCI-sponsored studies to address gaps in knowledge and the benefits and harms of cannabis and cannabinoid use in patients with cancer. Barriers and themes from the survey related to conducting research in this therapeutic area are discussed.

**Conclusions:**

Longitudinal studies evaluating the benefits and harms of cannabis use remain scarce. Significant gaps in knowledge persist for both providers and patients, compounded by regulatory, ethical, and drug provision hurdles in this research area. Our survey results offer a foundation for educating care team members about cannabis use. Alongside participation in a large, first-ever national study, we outline plans for a small pilot study that uses an innovative application to capture cannabis use and product type.

## 1. Introduction

As of November 2024, 24 states and the District of Columbia have legislated both medical and recreational (adult) cannabis access and use, another 17 states have legislated medical cannabis only, and 9 had no such legislation. ^1^ Remarkably, this legislative posture translates to 74% of Americans living in states where cannabis is legal for either medical or recreational use, and 79% of Americans living in a county with at least 1 cannabis dispensary. ^2^ In 1999, Maine was the sixth state in the nation to approve medical cannabis use after California’s lead in 1996. In 2016, Maine approved recreational cannabis use along with 4 other states, bringing the total to 8 states led by Washington and Colorado in 2012. ^1^ The craft cannabis industry in Maine is robust, with 154 registered recreational shops, 65 medical dispensaries, and an estimated 289 medical storefronts collectively generating more than $243.9 million in retail sales. ^3,4^

Between 20% and greater than 40% of patients with cancer use cannabis to relieve either symptoms of their disease or side effects of their treatment. ^5–10^ Cannabis use among patients with cancer is increasing and presents opportunities for both patients and providers to better understand and characterize the benefits and harms of cannabis use. ^11–13^ At the same time, both patients and providers need greater familiarity with various cannabis products, routes of administration, and drug content (eg, tetrahydrocannabinol [THC], the primary psychoactive component; cannabidiol [CBD], cannabigerol [CBG], and cannabinol [CBN], which are not intoxicating and among the 100+ cannabinoids found in the plant *Cannabis sativa*). ^14,15^ A recently reported national survey of 462 oncology fellow trainees sheds light on these challenges. ^16^ Of 462 respondents, 57% reported discussions with 5 or more of their patients about medical cannabis, 13% felt sufficiently knowledgeable, and 24% reported having received formal training. ^16^

In December 2020, the US National Cancer Institute (NCI) convened a first-ever 4-day conference. After the conference, the NCI published a dedicated symposium monograph on the role of cannabis and cannabinoids in oncology care entitled, *The Trans-National Institutes of Health Cannabis, Cannabinoids, and Cancer Research Symposium*. ^11^ A focus of the symposium was research opportunities and challenges on cannabis use in patients with cancer. Shortly thereafter, in October 2022, NCI and the National Institutes of Health (NIH) published RFA-CA-22-052, which solicited applications “to address gaps in knowledge and build evidence of the types of products in use, frequency and dosage of use, and the benefits and harms of cannabis and cannabinoid use during cancer treatment.” ^17^ This solicitation led to the NCI funding a coordinating center and 4 to 5 longitudinal cohort projects aimed at exploring and documenting cannabis use in patients with cancer.

With this backdrop and our response to RFA-CA-22-052, we surveyed providers in the MaineHealth Cancer Care Network (MHCCN), headquartered in Portland, to learn their understanding of and perspectives on cannabis use in their patients. Given the short turnaround time to respond to NIH funding opportunities, we restricted our survey to MHCCN care team members with the most proximate and/or direct patient care, who were most likely to engage in discussions about cannabis. The MHCCN comprises 8 member organizations and 1 affiliate organization, encompassing more than 350 providers who give patient care for an analytic tumor volume of more than 7500 cancer cases annually. This number represents nearly 75% of the cancer burden in Maine. This report summarizes our cannabis landscape survey of front-line providers of cancer care and care team members in our cancer network. In this manner, we aimed to gauge the reception and feasibility toward proceeding with cannabis-focused clinical research projects.

## 2. Methods

A voluntary, less than 10-minute, 20-question electronic survey was distributed to front-line MHCCN providers of cancer care and care team members using our institutional licensed *Research Electronic Data Capture (REDCap)* database (Vanderbilt University, Nashville, TN). These individuals included physicians (eg, medical, surgical, and radiation oncologists; palliative care), advanced practice professionals, nurses and nurse navigators, pharmacists, social workers, and practice managers at each MHCCN location. Some care team members of the MHCCN were not solicited to participate because they are less likely to discuss cannabis use with patients with cancer. These members included primary care physicians, specialty physicians involved in cancer screening, pathologists, radiologists, clinical trials office personnel, and laboratory scientists. The survey was conducted over 2 months, from December 2022 to January 2023, and was distributed via a link in an email sent by the program manager or NCI Community Oncology Research Program (NCORP) administrator. The email with the survey link outlined details of the intention to respond to the RFA-CA-22-052 and conveyed that the survey was confidential and no respondent would be identified. Providers were asked to share their professional degree/credential and MHCCN hospital or practice location, and they were not required to answer any question with which they were uncomfortable or did not want to answer. No personal or identifying information was recorded. Before distributing the survey, permission was granted from the MaineHealth Institutional Review Board.

## 3. Results

The survey was distributed to 171 front-line providers and care team members across 20 MaineHealth Cancer Care Network hospitals and cancer practices. Of these, 100 responded, yielding a 58% response rate (100/171). Respondents included 36 physicians (36/100), 25 nurses and nurse navigators (25/100), 16 pharmacists (16/100), 14 advanced practice professionals (14/100), 6 social workers (6/100), and 3 practice managers (3/100). The top 3 practice locations by number of solicitations were MHCC–Scarborough (36/171), MH Maine Medical Center (22/171), and MHCC–South Portland (19/171). Together, these locations accounted for 45% of all solicitations (77/171) (Table 1).

Table 2 summarizes the survey responses to all 20 questions. Important themes emerged from the survey. First, 30% of providers/care team members (ie, Question [Q]1–always and usually) inquire about cannabis use in their patients. Second, 89% (Q5) strongly and somewhat agree that cannabis can be effective for managing symptoms. Third, 54% are sensitive (Q7–strongly and somewhat agree) to stigma surrounding use and believe (Q8–strongly and somewhat agree) that 57% of their patients are sensitive to stigma as well. Fourth, only 15% (Q12) considered themselves knowledgeable in cannabis use. Fifth, 13% (Q17) refer patients to an integrative medicine physician or provider that is known to prescribe medical cannabis. Finally, 85% (Q18–yes and maybe) are receptive to learning more about cannabis for symptom management.

## 4. Discussion

The results from our survey revealed the willingness to participate in and advocacy for proceeding with 2 NCI research proposals exploring cannabis use in patients with cancer. The 58% response rate signals awareness and interest in cannabis use in front-line care team providers in our cancer practices. These observations, for the most part, align with a published survey of the Society of Cannabis Clinicians, a group that would be considered experienced and knowledgeable in prescribing and counseling their patients on cannabis use. ^18^ In this survey, 133 participants were assessed for eligibility, and 45 physician surveys were analyzed for a response rate of 34%. ^18^ The major discrepancy between their and our survey is that in their survey, 78% (29/37) of physician responses to a specific knowledge survey question considered themselves knowledgeable about cannabinoids and the endocannabinoid system, whereas in our survey, only 15% considered themselves knowledgeable. Also, only 13% of front-line providers in our survey referred patients to a physician or provider who was knowledgeable and known to prescribe medical cannabis.

As the prevalence of cannabis use increases in contemporary cancer practice, providers must become better informed about its use and safety profile, provide instruction and education during the formative years of oncology training, and help patients to feel comfortable disclosing and discussing use with their care teams. ^11–13,16,19^ A recently published, large retrospective case series in 3148 Australian patients using medical cannabis echo these thoughts. ^20^ Findings from this study suggest sustained benefits in health-related quality of life. However, and importantly, adverse events were common but rarely severe. ^20^ Recent clinical guidance from the American Society of Clinical Oncology and policy statements from the American College of Physicians advocate for more prospective clinical research, training, and efforts to address challenges with current federal and often conflicting state legislative frameworks. ^21,22^ NCI also advocates for advancing research in this area, given the paucity of well-designed prospective clinical studies exploring the benefits and harms of cannabis use. ^23,24^ Undoubtedly, hurdles persist, but chief among those regarded by the National Institute on Drug Abuse, NIH, US Food and Drug Administration (FDA), and researchers include the US regulatory status of cannabis and cannabinoids, sources for cannabis and cannabinoid study medications, and limited funding and resources to support studies. ^24^ After the release of RFA-CA-22-052, the NCI awarded 5 highly coordinated, longitudinal cohort studies in patients with newly diagnosed cancer to explore the benefits and harms of cannabis in thousands of patients under a cooperative U01-funding mechanism. These studies are summarized in Table 3 and have all been launched.

Maine is a small state (population of 1.38 million), is the most rural state (61.4% of the population resides in rural areas per the Health Resources and Services Administration criteria), and has the oldest population in the nation (median age of 44.8 years, 6 years older than the national median). ^25–28^ Given this profile, and Maine’s permissive legislative posture toward medicinal and recreational cannabis use and vibrant cannabis industry, the state is an ideal clinical laboratory to pursue research in this evolving therapeutic area. MHCCN investigators are participating in Wake Forest’s U01-supported Complementary Options for Symptom Management in Cancer (COSMIC) study (WF-2304-A1724014) as a rural site champion. ^29^ This joint Wake Forest NCORP Research Base and Alliance–led study aims to recruit 2000 patients with newly diagnosed breast, non-small cell lung, and colorectal cancer; melanoma; and non-Hodgkin lymphoma. These patients will be followed over 12 months. Patients will be recruited in 2 cohorts during cancer treatment: those using and not using cannabis in the context of pursuing other complementary therapies during treatment. Surveys documenting cannabis use, and the benefits and harms of use on cancer and treatment-related symptoms, will be collected. A subset of patients with lung cancer will participate in pharmacokinetic and pharmacodynamic correlative studies exploring drug-drug interactions and immunosuppressive effects of cannabis.

Our team developed a companion study to the COSMIC study, supported by a Wake Forest NCORP Research Base supplemental award. We launched an investigator-initiated study (COSMIC Releaf^™^, MH IRB# 2278577) in 45 patients with cancer who use cannabis. ^30^ Eligible patients are either (1) undergoing active treatment, including surgery, radiation, and systemic therapy as a single or combined modality therapy; or (2) in active follow-up after treatment or at any point along the survivorship spectrum. We focused on a stated objective outlined in RFA-CA-22-052 to document the precise product and drug content among patients that use cannabis, with the understanding that usually patients and providers do not know this information at the time of acquiring a drug/product and/or subsequent use. With this goal in mind, we are using a customizable, protocol-specific patient application, *Releaf App*^™^ (MoreBetter Ltd., Hyattsville, MD), that is suited for iPhone or Android platforms and supports journal entries to record cannabis use and the type of product. This application has been used by more than 18123 study participants across numerous studies and in a current FDA-sponsored study. ^31–40^ As part of the study start-up, we will work with the Releaf App to verify the product inventory at Maine cannabis storefronts’ [both medical-use and adult-use (recreation) stores] in the app. Thus, for participants, data on the type of cannabis and/or cannabinoid product, composition (eg, THC and CBD/CBG/CBN), and frequency of use will be directly captured in the app and linked to the storefront at the time of purchase. There are 2 project-specific electronic data capture systems for this project: REDCap and Penzai (US patent pending). Penzai is a web-based software application (MoreBetter, Ltd., Hyattsville, MD) that permits customizable, decentralized, and remote data collection. This software can also send (via text or email) reminders to participants on a pre-arranged schedule to prompt journaling of cannabis consumption in the Releaf App, which is highly suited to our study. These reminders will be sent weekly with formal monthly check-ins over 6 months of study follow-up. Nearly two-thirds of respondents in our survey valued learning more about cannabis use for patients’ symptoms management. Given this finding, the 2 studies we are participating in provide an excellent departure point to further expand on this opportunity. Our approach is innovative, and in this manner, we have every expectation to precisely capture the benefits and harms along with the cannabis product and content.

As we embarked on this study, we worked closely with our IRB on several aspects of our study and design. Chief among them is to ensure confidentiality and privacy of participants given the current legal environment regarding cannabis use. Two themes emerged. First, to protect patient confidentiality, we have been granted a waiver of documentation of written consent. The patient will be given an IRB-approved informed consent document to review with the study coordinator, who will document verbal consent and sign the form. The patient will retain this copy of the consent document, which will only include their study identification number. This arrangement avoids a signed consent document linking a patient to the study. A second theme is to make study participation agnostic to the electronic health record and, thus, ensure patient privacy regarding professional, employment, health insurance, or personal concerns. To accommodate data management, we will use 2 REDCap databases. One database will act as the master key database that holds protected health information and manages small stipends that have been incorporated into our study design to facilitate study participation. The other will hold study data. The master key database will be deleted at the end of the study. We have adopted these safeguards to enhance patients’ willingness to participate in our study. These steps are additional considerations we believe are important to address the inherent barriers in cannabis-related clinical study.

It is important to acknowledge that our survey was extended to approximately 50% of MHCCN care team members (n = 171) among more than 350 providers. This subset is most likely to engage in discussions with their patients about cannabis use that otherwise are unlikely with primary care physicians, radiologists, and pathologists, among others. This subset could be considered a limitation of our study. Furthermore, the response rate of 58% is robust for this type of survey and representative of MHCCN practice locations and front-line providers. Upon perusal of Tables 1 and 4, the response rate corresponds to the analytic tumor volume at each MHCCN practice, with one exception. The 2% response rate at the Harold Alfond Center for Cancer Care (Maine General Medical Center) does not correspond to the 17% analytic volume that this location contributes to the network. In short, there is good representation of provider input across metropolitan, micropolitan, and rural practices that adds value.

Lastly, 3 important themes from our survey provide opportunities for future care team learnings and engagement. Notably, only 15% of respondents are knowledgeable in cannabinoids and the endocannabinoid system, so there is considerable opportunity for providing educational platforms for our providers, especially given the growing prevalence of cannabis use by patients with cancer. ^19,21,22^ Similarly, it is important to incorporate instruction in our hematology/oncology fellowship program that is a gap nationally. ^16^ And although there is integrative medicine and cannabis expertise locally, referral channels can be enhanced to capitalize on this resource.

## 5. Conclusions

Medicinal and recreational cannabis and cannabinoid use in patients with cancer has rapidly scaled up over the past decade and is a topic of considerable contemporary clinical and research relevance, and public health interest. There is a paucity of longitudinal studies and clinical research evaluating the benefits and harms of cannabis use. There are gaps in knowledge for both providers and patients on the pharmacokinetic (especially drug-drug interactions) and pharmacodynamic effects of this class of compounds in the clinic. There are inherent regulatory, ethical, and drug provision hurdles, among others, in this clinical research area. However, NIH, other health regulatory agencies, and professional societies highly support actively pursuing this line of clinical investigation into the use of cannabis. The NCI is taking the lead on an initial series of large prospective cohort studies exploring the scale and scope of cannabis use in patients newly diagnosed with cancer. We describe our plans for a small pilot investigation using an innovative application to capture cannabis use and the type of product consumed. Lastly, there is value in developing educational opportunities for our providers as well.

## Figures and Tables

**Table 1.** Invitations and responses rate to the cannabis landscape survey by hospital and practice location.

MH hospital	Providers invited to participate (n = 171)	Response rate relative to total respondents, % (n = 100)
**Metropolitan area**		
MH Maine Medical Center (Portland)	91	57
MD	36	25
APP	14	9
RN	15	12
PharmD	19	7
Other	7	4
**Micropolitan areas**		
Maine General - Harold Alfond Center for Cancer Care (Augusta)	16	2
MD	3	2
APP	0	0
RN	8	0
PharmD	1	0
Other	4	0
MH Mid Coast Hospital (Brunswick)	11	6
MD	4	3
APP	2	2
RN	1	0
PharmD	2	0
Other	2	1
St. Mary’s Regional Medical Center (Lewiston)	6	3
MD	1	0
APP	1	1
RN	2	2
PharmD	1	0
Other	1	0
MH Maine Medical Center (Biddeford-Sanford)	19	14
MD	4	4
APP	0	0
RN	6	4
PharmD	7	4
Other	2	2
**Rural areas**		
MH Franklin Hospital (Farmington)	3	3
MD	0	0
APP	0	0
RN	2	2
PharmD	1	1
Other	0	0
**Metropolitan area**		
MH Lincoln Hospital (Damariscotta)	3	1
MD	0	0
APP	0	0
RN	0	0
PharmD	2	0
Other	1	1
MH Memorial Hospital (North Conway, New Hampshire)	3	1
MD	0	0
APP	1	0
RN	0	0
PharmD	1	0
Other	1	1
MH Pen Bay Hospital (Rockport)	7	5
MD	1	1
APP	2	1
RN	0	0
PharmD	2	1
Other	2	2
MH Stephens Hospital (Norway)	5	4
MD	0	0
APP	1	1
RN	2	1
PharmD	0	0
Other	2	2
MH Waldo Hospital (Belfast)	7	4
MD	1	1
APP	1	0
RN	1	1
PharmD	2	0
Other	2	2
**Total**	171	100
MD	50	36
APP	22	14
RN	37	23
PharmD	38	13
Other	24	14

Abbreviations: APP, advanced practice provider; MD, medical doctor; MH, MaineHealth; PharmD; doctor of pharmacy; RN, registered nurse.

**Table 2.** MHCCN Cannabis Landscape Survey Questions and Responses.

Question	Response rate, %*
(1) Do you ask patients about marijuana/cannabis use? (n = 100)	
Always	14
Usually	16
Sometimes	38
Not often	22
Never	10
(2) Do you document in the electronic health record if a patient uses marijuana/cannabis? (n = 100)	
Yes (n = 69)	
Office note element (n = 38)	55
Social history (n = 19)	28
Medication list (n = 12)	17
No (n = 31)	
Professional concerns for patient (n = 4)	13
Patient request (n = 3)	1
Legal concerns (n = 1)	<1
Employment concerns for patient (n = 0)	0
Other (n = 23)	74
(3) Patients are forthcoming about using marijuana/cannabis? (n = 100)	
Strongly agree	12
Somewhat agree	57
Neither agree nor disagree	21
Somewhat disagree	6
Strongly disagree	4
(4) If patients are forthcoming about their marijuana/cannabis use, they are satisfied and comfortable with managing their symptoms with its use. (n = 100)	
Strongly agree	18
Somewhat agree	42
Neither agree nor disagree	40
Somewhat disagree	0
Strongly disagree	0
(5) Medical cannabis is (or can be) effective for managing clinical symptoms (such as pain, nausea/vomiting, insomnia, and anxiety). (n = 100)	
Strongly agree	42
Somewhat agree	47
Neither agree nor disagree	10
Somewhat disagree	1
Strongly disagree	0
(6) Patients are comfortable approaching the subject of medical cannabis with me. (n = 100)	
Strongly agree	19
Somewhat agree	41
Neither agree nor disagree	31
Somewhat disagree	9
Strongly disagree	0
(7) Providers are sensitive to stigma surrounding the medical usage and recommendations of cannabis. (n = 100)	
Strongly agree	13
Somewhat agree	41
Neither agree nor disagree	38
Somewhat disagree	7
Strongly disagree	1
(8) Patients are sensitive to stigma surrounding medical usage of marijuana/cannabis. (n = 100)	
Strongly agree	10
Somewhat agree	47
Neither agree nor disagree	31
Somewhat disagree	10
Strongly disagree	2
(9) If I felt educated on its use, I would prescribe medical marijuana to my patients if it were federally legal and permissible within my practice/organization. (n = 100)	
Strongly agree	32
Somewhat agree	20
Neither agree nor disagree	25
Somewhat disagree	8
Strongly disagree	6
Unsure at this time	9
(10) What are some of the symptoms that patients’ use or would like to use marijuana/cannabis for? (Please check all that apply.) (n = 78)	
Anxiety (n = 78)	100
Decreased appetite (n = 78)	100
Nausea/Vomiting (n = 75)	96
Pain, general (n = 68)	87
Insomnia (n = 67)	85
Pain, neuropathy (n = 45)	58
Depression (n = 36)	46
Other (n = 4)	5
Unsure – I do not have this discussion (n = 11)	14
(11) Briefly explain your recommendations when a patient asks about marijuana/cannabis use to alleviate symptoms associated with cancer. (n = 98)	Most common responses: • Consuming edibles preferred over smoking or inhalation of cannabis. • Seeing their oncology provider or primary care physician. • Education on what is known about risks and benefits. • Speaking with a medical provider that prescribes marijuana cards and is also knowledgeable about cannabis products and dosages for patient use.
(12) Do you consider yourself knowledgeable about marijuana/cannabis methods of use and types of products? (n = 99)	
Yes	15
No	36
Somewhat	48
Did not answer	1
(13) Do you consider yourself knowledgeable about cannabis THC/CBD content? (n = 100)	
Yes	9
No	50
Somewhat	41
(14) Do you consider yourself knowledgeable about marijuana/cannabis laws in Maine? (n = 99)	
Yes	14
No	33
Somewhat	52
Did not answer	1
(15) Do you consider yourself knowledgeable about the biologic effects in the body from marijuana/cannabis use? (n = 98)	
Yes	16
No	30
Somewhat	52
Did not answer	2
(16) Do you consider yourself knowledgeable in cannabis use disorder? (n = 100)	
Yes	12
No	51
Somewhat	37
(17) Do you refer patients to an integrative medicine physician or provider that is known to prescribe medicinal marijuana/cannabis? (n = 98)	
Yes	13
To whom did you refer	• Integr8 Health^†^ • Palliative care • Medical oncology/Primary care physician
No	85
Did not answer	2
(18) Are you interested in learning more about marijuana/cannabis use for patients’ symptom management? (n = 99)	
Yes	64
No	14
Maybe	21
Did not answer	1
(19) Would you like a resource/source of information identified by an integrative medicine physician specializing in medical marijuana/cannabis use to refer patients with cancer? (n = 99)	
Yes	71
No	9
Maybe	19
Did not answer	1
(20) If MHCCN is awarded project funding, would you be comfortable referring patients to the study coordinator? (n = 99)	
Yes	77
No	3
Maybe	19
Did not answer	1

Abbreviations: CBD, cannabidiol; MHCCN, MaineHealth Cancer Care Network; THC, tetrahydrocannabinol.

* Unless otherwise indicated.

† Locoregional integrative medicine practice in southern Maine.

**Table 3.** Awards Made in Response to RFA-CA-22-052*.

Institution	Grant #	Project title
City of Hope	5U01 CA286808	A prospective cohort study of patients with non-small cell lung cancer and multiple myeloma to assess the benefits and harms related to cannabis use during treatment
Georgetown University	5U01 CA286821	Longitudinal assessment of benefits and harms of cannabis use among community-based cancer patients during initial cancer treatment
University of Buffalo	5U01 CA286811	Assessing benefits and harms of cannabis use in patients treated with immunotherapy for cancer: a prospective cohort study
University of Miami	5U01 CA286810	Assessing benefits and harms of medical cannabis and cannabinoid use in breast cancer patients during and after treatment
Wake Forest University	5U01 CA286813	Assessing benefits and harms of cannabis and cannabinoid use among a cohort of cancer patients treated in community oncology clinics

* Available through NIH RePORTER (https://reporter.nih.gov) search by entering the CA-6-digit grant number.

**Table 4.** Analytic Tumor Volume of the MHCCN by Hospital and Practice Location.

MH hospital*	2022 analytic tumor volume,^†^ No. (%)
Metropolitan area	
MH Maine Medical Center (Portland)	3886 (51.4)
Micropolitan areas	
Maine General - Harold Alfond Center for Cancer Care (Augusta)	1270 (16.8)
MH Mid Coast Hospital (Brunswick)	406 (5.4)
St. Mary’s Regional Medical Center (Lewiston)	229 (3.0)
MH Maine Medical Center (Biddeford-Sanford)	618 (8.1)
Rural areas	
MH Franklin Hospital (Farmington)^‡^	166 (2.2)
MH Lincoln Hospital (Damariscotta)^‡^	106 (1.4)
MH Memorial Hospital (North Conway, New Hampshire)^‡^	171 (2.3)
MH Pen Bay Hospital (Rockport)	416 (5.5)
MH Stephens Hospital (Norway)^‡^	136 (1.8)
MH Waldo Hospital (Belfast)^‡^	156 (2.1)
Total	7560 (100)

Abbreviations: MH, MaineHealth; MHCCN, MaineHealth Cancer Care Network.

* Population service area per definition of Core Based Statistical Areas.

† Cancer Registry 2023 Annual Report. Maine Medical Center and MaineHealth Cancer Care Network. MaineHealth, Portland, ME.

‡ Critical access hospital.
